# Impact of smart locker use on customer satisfaction of online shoppers in Vietnam

**DOI:** 10.1057/s41599-022-01428-6

**Published:** 2022-11-04

**Authors:** Nguyen Hong Quan, Nguyen Thi Binh, Bui Thi Ly

**Affiliations:** Foreign Trade University, Hanoi, Vietnam

**Keywords:** Business and management, Information systems and information technology, Business and management

## Abstract

In recent years, smart lockers have been increasingly utilised in the last-mile delivery stage of e-commerce. Although this technological advance has received significant scientific attention, especially in developed countries, little is known about the general behaviour of Smart Locker users. This study aimed to explore the impact of smart locker use on customer satisfaction with online shopping experience. We used structural equation modelling to analyse data collected from 442 smart locker users in Vietnam. The results revealed that convenience, privacy and security, and reliability attributes significantly impacted the satisfaction of smart locker users through the mediators of perceived value and transaction costs. These findings are expected to reinforce the utilised theories and enrich theoretical research on self-service technologies in last-mile delivery and their influence on consumer behaviour.

## Introduction

In the context of rapid e-commerce growth, e-retailers must pay more attention to customer experience to gain a competitive edge (Pine and Gilmore, 2011; Verhoef et al., 2009). Consequently, a significant amount of research has been conducted to explore the determinants and tools that affect customer satisfaction in online shopping (Jun et al., 2004; Liu et al., 2008; Alam and Yasin, 2010; Guo et al., 2012; Wu, 2013; Shahin et al., 2014; Vasić et al., 2019). Last-mile delivery significantly impacts customer satisfaction in an online shopping environment (Liu et al., 2008; Alam and Yasin, 2010; Guo et al., 2012; Vasić et al., 2019; Rahi et al., 2020). The quality of the delivery service is usually reflected in measurement items, such as delivery reliability, delivery time, package safety, shipping costs, with timeliness and reliability commonly recognised as the most appreciated factor by customers (Ziaullah et al., 2014; Vasić et al., 2020). Novel delivery solutions have emerged to reduce the increasing pressure on e-commerce logistics, particularly on last-mile delivery, and improve customer satisfaction (Ferrucci and Bock, 2014). Smart Locker is a technology that received ample attention from e-commerce businesses, logistics service providers, and scholars.

Smart Lockers are self-service pick-up lockers that provide 24-hour service and allow users to collect their goods flexibly (Mostakim et al., 2019). They offer customers a new and simple last-mile delivery method that requires no on-site staff operation (Montreuil, 2016). Several benefits of this delivery service have been identified. It eliminates home delivery failure and redelivery and takes advantage of economies of scale by collecting and delivering the goods to one location, thereby reducing last-mile delivery costs (Zenezini et al., 2018; Van Duin et al., 2020). It has a positive impact on the environment by minimising traffic congestion, noise pollution, and greenhouse gas emissions due to fewer delivery trips (Chen et al., 2018; Ranieri et al., 2018). It allows customers to pick-up goods in their most favourable conditions, without the need to wait at home for a delivery or depend on the courier (Djelassi et al., 2018). However, most studies have focused on developed e-commerce markets with larger size and higher growth rates, such as China and Sweden, whereas information on emerging e-commerce markets, such as Vietnam, is scarce. Furthermore, to the best of our knowledge, existing research has not considered the impact of using Smart Lockers on e-customer satisfaction. This topic merits investigation, as last-mile delivery methods may affect e-customer satisfaction differently. Therefore, this study aimed to explore the relationship between Smart Locker use and customer satisfaction with online shopping experience in Vietnam. We proposed a model for impact assessment of Smart Lockers on customer satisfaction to identify differences between emerging and established e-commerce markets. The study is expected to enrich the theoretical and empirical understanding of the impact of last-mile delivery on consumer behaviour and offer e-commerce businesses a new perspective on the role of last-mile delivery in enhancing customer satisfaction.

## Theoretical framework

### Smart Locker technology

Smart Lockers are customisable and scalable electronic systems that leverage cloud computing to provide users discernible rooms for the collection of documents or packages (Yuen et al., 2019). Smart Lockers can be understood as automatic storage lockers that do not need to be operated or managed by employees. They are fixed in a safe location outside the customer’s residence, such as ground floor of an apartment building, workplace, parking lot, and train station, and can be opened with a key or electronic code. Smart Lockers are largely used in parcel delivery services; however, they can also be applied to the food delivery sector if temperature control devices are included (Iwan et al., 2016).

Therefore, Smart Lockers are a type of self-service technology (SST), where the customer is partly responsible for the value creation process (Svensson and Grönroos, 2008) and can also be viewed as a service provider (Meuter et al., 2000). Smart Lockers created a new B2C distribution channel, offering customers a simple delivery option that requires no on-site staff operations (Montreuil, 2016). Focusing on the reduction of failed deliveries due to consignee not showing up or refusing to pick up, and leveraging opportunities for consolidated shipping, Smart Lockers are increasingly seen as a promising solution for last-mile deliveries and return.

### Customer satisfaction with self-service technology

Determinants of customer satisfaction with SST have received increasing attention since the early 2000s. Various theoretical frameworks were utilised to analyse the potential sources of satisfaction (Table 1).

**Table 1:** Critical studies on customer satisfaction with self-service technology.

Source	Sector—country	Theoretical framework	Method	Key points
Wang (2012)	Convenience store chain—Taiwan	The Expectation—Confirmation Model in the context of IT (Bhattacherjee, 2001)	Structural equation modelling	Antecedents of SST satisfaction are (1) perceived usefulness, (2) perceived enjoyment, (3) perceived control and (4) perceived convenience. Elements (1) & (2) impact (3) & (4) and then consumer satisfaction. In addition, while (2) has a positive impact on consumer satisfaction, (1) does not.
Boon-itt (2015)	Digital banking—Thailand	The Technology Acceptance Model (Davis et al., 1989) and the SST adoption model (Bitner et al., 2000)	Structural equation modelling	Technology readiness acts as an antecedent that influences the service quality of SST, which in turn affects e-satisfaction. Even though the service quality of SST positively influences e-satisfaction, perceived value mediates the level of impact.
Djelassi et al. (2018)	Mass retail chain—France	An original mediation model	Structural equation modelling	Waiting time satisfaction mediates the relationship between SST experience evaluation and its satisfaction, then significantly mediates the effect of SST experience evaluation on the customer satisfaction towards the retail store.
Tang et al. (2021)	e-Commerce—China	The SERVQUAL model (Parasuraman et al., 1988), the logistics service quality evaluation model (Mentzer et al., 2001), and the modified model of e-service quality (Raza et al., 2020)	Confirmatory factor analysis	The service quality of IoT-based smart parcel lockers is assessed on five dimensions: price, reliability, convenience, fault handling capability, and diversity, among which service price does not have a positive impact on consumer satisfaction.

Source: Compiled by the author.

Previous research has examined the antecedents of SST satisfaction and factors mediating their impact on the level of satisfaction. Direct or indirect determinants can be divided into two groups: service quality attributes (functional value) and perceived value (emotional value). Djelassi et al. (2018) demonstrated the mediating effects of SST satisfaction on the relationship between SST experience evaluation and customer satisfaction in retail.

### Consumer theories

In the domain of information systems, customer satisfaction is the key driving factor in the continuance of repurchase intention (Churchill and Surprenant 1982; Bhattacherjee, 2001; Wang, 2012; Djelassi et al., 2018). Yuen et al. (2019) integrated the theories of resource matching, perceived value, and transaction cost to create a model to evaluate customers’ intention to repeatedly use Smart Lockers for last-mile delivery. All three theories were applicable to explain customer satisfaction. The combination of the theories provided a more comprehensive approach to examine customers’ post-use evaluation of their experience with Smart Lockers.

The resource matching theory offers insights into how Smart Locker attributes could lower users’ perceived effort and improve satisfaction with the experience. SSTs require various resources from customers, such as time, money, and cognitive and physical effort (Collier and Kimes, 2013). While individuals’ available resources are dependent on their motivation and ability, what they perceive as necessary can be altered through the design and management of the service (Chen et al., 2018).

The perceived value theory, which can be seen as an extension of the resource matching theory, supports the argument that the efficient matching of resources, influenced by Smart Locker attributes, including convenience, privacy and security, and reliability, promotes customers’ perceived value and improves service satisfaction. A multi-dimensional scale has been applied to measure the perceived value of Smart Locker use. The scale consists of three utility dimensions: functional, such as utility and performance, hedonic, such as feelings and affection, and social, such as positive externality (Sweeney and Soutar, 2001; Wang et al., 2004; Yuen et al., 2019). This study omitted the economic value dimension, as customers were not surcharged for using Smart Lockers in the surveyed services.

The transaction cost theory, which can also be seen as an extension of the resource matching theory, posits that transaction costs reflect the complexity of an exchange: the higher the complexity, the higher the costs (Williamson, 2008). The present study used the transaction cost theory to explain how better resource matching reduces transaction costs, proposing that consumers do not evaluate a service solely on its price or benefits but also on the opportunity costs incurred as a result of using that service. Two essential indicators of transaction costs are perceived ease of use and time efficiency (Devaraj et al., 2002). We measured transaction costs as the costs of searching, learning, and effort involved in Smart Locker use (Tate et al., 2011; Yuen et al., 2019).

### Hypothesis development

#### The effects of convenience, privacy and security, and reliability on perceived value and transaction costs

Smart Locker hubs placed within close vicinity of customers’ domicile, workplace, and traffic intersections are geographically more convenient for those who commute (Yuen et al., 2019). Furthermore, customers can retrieve their packages from Smart Locker hubs at any convenient time in lieu of waiting at home for the courier (Chen et al., 2018). Moreover, in the era of digitalisation, customers are more prone to accept and quickly respond to new technologies, especially if they are smartphone-based (Turkle, 2017). Consequently, the effort required to learn and use Smart Lockers is low, as information, such as delivery time, OTP code, instructions, and policies, is exchanged through mobile devices. Thus, this study proposed that convenience can enhance customers’ perceived value through improving functional (i.e., reduced unproductive use of time and decreased effort) and emotional (i.e., creation of excitement and enjoyment) utility.

##### H1a: Smart Locker convenience positively affects customers’ perceived value.

Smart Locker hubs located near customers’ residence or locations they frequent for daily tasks have the ability to reduce the amount of time and money required for travelling, resulting in lower transaction costs (Liu et al., 2019). In addition, Smart Lockers improve time efficiency by eliminating unnecessary waiting commonly present in door-to-door delivery and offering round-the-clock accessibility, which in turn reduces transaction costs. Furthermore, by virtue of the smart and user-friendly design, Smart Lockers require low transaction costs in the process of learning and using the service (Alaghehband et al., 2011).

##### H2a: Smart Locker convenience negatively affects customers’ transaction costs.

In comparison with home delivery or collection points, such as stores in the PostCo system employed by Lazada, Smart Lockers have the advantage of requiring no human-to-human interactions (Featherman et al., 2010). This unique trait protects customers against the risk of exposing private information, such as phone numbers and addresses, to logistics companies’ employees. All personal data are encrypted in the system and customers are asked to authenticate their identity to gain access to the parcels. Conversely, security policies, the function of which is to advise customers of the way a company would treat their personal data, provide critical insights into the service provider’s security system (Xu et al., 2011). Such policies can positively contribute to enhancing customers’ trust and minimising concern over privacy violations (Chua et al., 2017).

Therefore, privacy and security increases customers’ perceived value regarding functional (i.e., through value co-creation and perceived level of security provided), hedonic (i.e., a sense of trust and safety), and social (i.e., better compliance with social norms and general demand for information security) utility (Lee and Lyu, 2016; Wang and Lin, 2017).

##### H1b: Privacy and security positively affect customers’ perceived value.

Although security protocols, such as entering OTP codes and ID numbers and scanning QR codes, can potentially complicate the interaction with the interface, these features encourage trust from customers, thus reducing perceived security risks associated with the use of Smart Lockers (Wang and Lin, 2017). Improvements in privacy and security directly and indirectly lower transaction costs. Costs can arise from dealing with the aftermath of security issues, such as identity theft, misuse of personal data, unauthorised access of users’ account, and security system breaches; however, they can also manifest in the form of unnecessary precautions, such as time and effort spent researching the security system, assessing the risks of handing over information to that system, and adopting preventive measures against potential violation (Song et al., 2016; Yuen et al., 2019).

##### H2b: Privacy and security negatively affect customers’ transaction costs.

Smart Lockers minimise the number of unsuccessful deliveries and are considered more reliable compared to home delivery (Faugere and Montreuil, 2017). Smart Locker customers receive notices from the logistics operator once the parcels have reached the designated hub and are ready for retrieval. Humans are predisposed to errors regarding knowledge, operation, and judgement, creating an increasing need for an automated structure capable of minimising manual control and enhancing last-mile logistics reliability (Chang and Wang, 2011). These traits heighten customers’ perceived value of the functional (i.e., an error-free service encounter) and hedonic (i.e., a more enjoyable experience) aspects (Yuen et al., 2019).

##### H1c: Smart Locker reliability positively affects customers’ perceived value.

Smart Lockers can reduce avoidable transaction costs caused by delayed or unsuccessful delivery (Akeb et al., 2018). Potential sources of costs may be the customers’ specific situation, such as time and effort wasted waiting for the courier, delaying and rescheduling the delivery, and asking another person to take temporary custody of the parcels, or their interactions with the transport operator, such as time and effort spent on retrieving misdelivered packages and contacting customer service.

*H2c: Smart Locker reliability negatively affects customers’ transaction costs*.

#### The mediating effects of perceived value and transaction costs on customer satisfaction with Smart Lockers

Koo (2006) used the means-end chain theory and suggested that customers’ perceptions of value guide their judgement on relevant attributes and service. Consequently, if customers recognise the amount of utility obtained from using the service as improving or helpful to achieve their personal values (i.e., instrumental and terminal values), they develop positive feelings toward the service. The idea that perceived value mediates the effect of perceived quality on customer satisfaction aligns with one of the central hypotheses of the Customer Satisfaction Index model (Hsu, 2008). In addition, perceived value has been empirically proven to have a positive impact on customer satisfaction in the context of SST (Boon-itt, 2015; Kim and Park, 2019; De Leon et al., 2020).

##### H3: Perceived value positively affects customer satisfaction with Smart Lockers.

According to Devaraj et al. (2002), any decrease in transaction costs, captured in time efficiency and perceived ease of use, leads to increased customer satisfaction. This means that when a rational customer incurs lower opportunity costs (i.e., time and effort) as a result of using a service, they will feel more satisfied with it. This idea has been empirically supported in prior studies (Jones and Leonard, 2007; Su et al., 2008). Therefore, the following hypothesis was proposed:

*H4: Transaction costs negatively affect customer satisfaction with Smart Lockers*.

#### The effect of customer satisfaction with Smart Lockers on the overall satisfaction with the e-commerce experience

Lemon and Verhoef (2016) developed a comprehensive model depicting customers’ journey, starting from the pre-purchase phase, flowing through purchase, and culminating in post-purchase. In the B2C context, it can be concluded that e-customers’ experience does not end until they gain physical possession of the goods. Jiang and Rosenbloom (2005) provide empirical evidence for changes in customer satisfaction at checkout and after delivery. Although this phenomenon could be blamed on the products’ failure to meet expectations, last-mile logistics, such as delivery reliability and consistency, are also critical. In addition, customers do not view retail experiences as separate encounters with different firms but rather as a connected overall experience created by a service delivery network made up of two or more organisations (Tax et al., 2013). Therefore, satisfaction with last-mile logistics is a variable dictating the outcome of overall satisfaction.

##### H5: Customer satisfaction with Smart Lockers positively affects the overall satisfaction with the e-commerce experience.

**Fig. 1:**
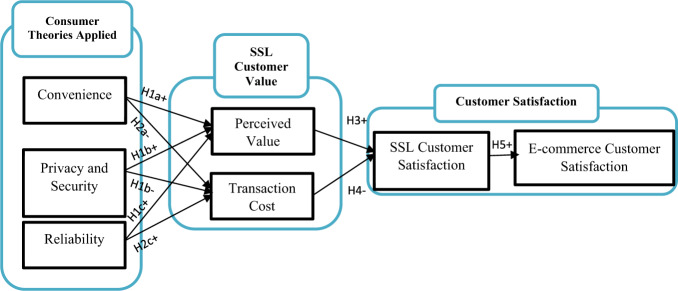
Conceptual framework model.

Overall, the conceptual framework model adopted in this study is presented in Fig. 1.

## Methods

This study used structural equation modelling (SEM) to analyse the impact of Smart Locker use on online shopping customer satisfaction. SEM is a powerful technique that can combine complex path models with latent variables (factors). Therefore, it fits our system of relationships between constructs rather than a dependent variable with a set of independent variables. Previous studies on online shopping and SST used Likert-scale analyses with SEM for path models (Porter and Donthu 2006; Yuen et al., 2019).

This study used covariance-based (CB)-SEM, as it is more appropriate for theory testing or confirmation, whereas partial least squares (PLS)-SEM is useful if the objective is for prediction of the endogenous variables (Jannoo et al., 2014). This was consistent with the purpose of our study, which was to test the hypotheses for the impact of factors related to Smart Locker use on customer satisfaction when buying online. Furthermore, PLS-SEM path estimates are less precise than CB-SEM under both normal and non-normal conditions when sample size is sufficient (Jannoo et al., 2014).

### Measuring observed variables

We proposed a measurement scale based on existing studies, which included 30 manifest variables corresponding to seven factors, to measure observed variables in the model. An expert panel discussion was employed to assess the relevance of the existing scale to Vietnamese users’ characteristics, thereby eliminating inappropriate items and introducing new ones (Table 2).

**Table 2:** Constructs and measurement items.

ID	Manifest variables	Sources of reference
Factor 1: Convenience		
CVN1	Smart Lockers are located close to users’ residence or daily activities	Lin and Hsieh (2011); Collier et al. (2014); Yuen et al. (2019)
CVN2	Locations of Smart Lockers are convenient to pick-up goods	
CVN3	Smart Locker allows users to pick-up goods at different times	
CVN4	Users can easily interact with Smart Locker to pick-up goods	
CVN5	Storage time in locker is enough for users to pick-up goods	
Factor 2: Privacy security		
PVT1	Smart Locker provides a safe using experience	Barua et al. (2018); Lin and Hsieh (2011); Yuen et al. (2019); Vakulenko et al. (2019)
PVT2	The user’s personal information is not used for unspecified purposes.	
PVT3	Users’ personal information is protected from the risk of theft	
PVT4	Smart Locker’s information security policy is clear, specific, detailed	Recommended by experts
Factor 3: Reliability		
REL1	Smart Locker provides the service as committed	Barua et al. (2018); Demoulin and Djelassi (2016); Yuen et al. (2019)
REL2	Smart Locker rarely has technical errors	
REL3	Smart Locker is more reliable than a delivery service made by humans	
REL4	Smart Locker helps to preserve goods well	Recommended by experts
Factor 4: Perceived value		
VAL1	Users feel pleasant using Smart Lockers	Collier et al. (2014); Yuen et al. (2019); Wang et al. (2020); Vakulenko et al. (2019)
VAL2	Users feel that using Smart Locker helps to pick-up goods quickly	
VAL3	Users find the pick-up experience with Smart Locker more enjoyable	
VAL4	Users feel that using Smart Locker has a positive impact on the environment and society	
Factor 5: Transaction cost		
TCT1	Users have to make a significant effort to search for Smart Locker related information	Yuen et al. (2019)
TCT2	Users have to make a significant effort to learn how to use Smart Lockers	
TCT3	Users have to make a significant effort to find a Smart Locker location	Proposed by the authors
TCT4	Users have to make a significant effort to physically travel to the Smart Locker location	Yuen et al. (2019); Vakulenko et al. (2019)
Factor 6: Customer satisfaction with Smart Locker		
SSL1	Users are satisfied with the experience of using Smart Locker	Vakulenko et al. (2019)
SSL2	Users feel Smart Locker exceeds their expectations	Recommended by experts
SSL3	Users will continue to use Smart Lockers	Vakulenko et al. (2019)
SSL4	Users will encourage others to use Smart Locker	
Factor 7: Customer satisfaction with E-commerce		
SEC1	Consumers are satisfied with the variety of service options offered when shopping online	Recommended by experts
SEC2	Consumers are satisfied with the online shopping experience	Vakulenko et al. (2019)
SEC3	Consumers will reuse online shopping services	
SEC4	Consumers will encourage others to use online shopping services	

The survey questionnaire was translated and calibrated in the Vietnamese language. Before conducting the survey, we conducted a pilot survey with 12 random Smart Locker users to ensure the clarity and straightforwardness of the questionnaire. Consequently, the final questionnaire was respondent-friendly.

The first section consisted of 14 questions, measuring the experience of using the Smart Locker service in three aspects: convenience, privacy and security, and reliability. The second section consisted of eight questions concerning perceived value and transaction costs. The third section consisted of eight questions, measuring user satisfaction with Smart Lockers and overall customer satisfaction with the online shopping experience that involved this logistics service. All items were measured using a five-point Likert scale (1 = strongly disagree; 5 = strongly agree).

In addition, questions regarding general information were included to provide a demographic profile of the data and a better understanding of users’ online buying habits (Table 3).

**Table 3:** Sampling criteria for SEM analysis.

Number of factors	Communalities value in EFA analysis	Minimum sample size
From 5 factors or less	From 0.6 and more	100
From 7 factors or less	From 0.5 and more	150
	From 0.45 and more	300
A large number of factors	About 0.4	500

Source: Hair et al. (2010).

### Sampling

The sample size was determined to ensure the standards for using exploratory factor analysis (EFA), confirmatory factor analysis (CFA), and SEM analysis and corresponded to the number of factors and observed variables of the proposed model.

Based on Hair et al. (2014), EFA consisting of seven factors and 30 manifest variables requires a sample size of 150 to 300. Based on Myers et al. (2011), the minimum sample size required for CFA is 300. Furthermore, according to Hair et al. (2014), SEM requires a sample size determined based on the number of factors and communalities value in EFA analysis.

With the mentioned characteristics of the proposed model, the minimum sample size was 150 (with the value of communalities when analysing EFA being from 0.5 or more) or 300 (with the value of communalities when analysing EFA being from 0.45). Thus, this study used a sample size of 500 to ensure that the conditions for performing the above analysis were satisfied.

### Data collection

The survey was conducted over a period of 30 days, from March 5, 2021 to April 4, 2021. The respondents were people who have used Smart Lockers when shopping online in Vietnam. During the investigation, we received strong support from a leading company in providing intelligent delivery solutions in Vietnam to reach the target respondents and ensure reliability.

To ensure the representativeness and reliability of the sample during the research process, as well as accessibility, we sent 500 questionnaires through three channels: 42% of the questionnaires were directly distributed at hubs in Hanoi, 18% were distributed via phone calls, and 40%, over the Internet. The study purpose, anonymity, and voluntary nature of participation were explained to the participants before responding to the survey. After screening, the dataset used in the study included 442 valid participant responses. The demographics of the sample are presented in Table 4.

**Table 4:** Sample descriptive statistics.

Characteristics		Frequency	Percentage
Gender	Male	242	54.8%
	Female	200	45.2%
Age	Under 18 years old	5	1.1%
	From 18 to 25 years old	334	75.6%
	From 26 to 35 years old	68	15.4%
	From 36 to 45 years old	33	7.5%
	Above 45 years old	2	0.5%
Education level	High school	13	2.9%
	Undergraduate	276	62.4%
	Graduate	99	22.4%
	Post-graduate	54	12.2%
Income	Under 5 million VND	234	52.9%
	From 5 to less than 20 million VND	115	41.2%
	From 20 to less than 30 million VND	10	2.3%
	Above 30 million VND	16	3.6%

The percentages of men and women were 54.8% and 45.2%, respectively. As Smart Locker is a relatively high-tech form of delivery in Vietnam, 76.7% of respondents were under the age of 26 years, whereas the oldest group, 46 years old or above, made up only 0.5%. Over half (62.4%) of the respondents were students, and the majority (78.9%) of the sample had an income of less than 10 million VND. Young people, including students and office workers, were assessed to be more receptive to new technological solutions, such as Smart Locker, and identified as the target customers. Therefore, Smart Locker hubs tended to be placed in universities, office buildings, and shopping malls in Vietnam.

## Results and discussion

This study applied SEM to evaluate the constructs and test the hypotheses through two main phases: (i) assessment of the measurement scale’s properties and (ii) discussion of the structural model.

### Measurement scale analysis

Churchill (1979) recommended conducting pilot tests of scale items generated from literature review to refine them using exploratory statistics. Therefore, after obtaining the conceptual framework from previous studies and consulting with experts, we conducted EFA analysis to determine whether the observed variables converged on the original scale of the study. Subsequently, we drew conclusions regarding whether the proposed conceptual framework (Fig. 1) was suitable. A finalised conceptual framework was developed based on the theories and verified by real data. A Cronbach’s alpha test was conducted to remove unusable items, which can create virtual factors, before performing EFA (Churchill, 1979).

EFA was performed with the Principal Axis Factoring method and Promax rotation for the model of 29 observed variables. Seven factors were extracted, corresponding to the number of factors included in the model, and each group of factors converged with the same observed variables as the original scale. As shown in Table 5, all observed variables had the absolute value of the loading factor greater than 0.5, and all latent variables had Eigenvalue coefficients greater than 1. According to Hair et al. (2010), this is the optimal level, showing that the observed variables have good statistical significance. This indicated that the seven latent variables should be kept in the model.

**Table 5:** Cronbach’s alpha, EFA, and CFA results.

Factors and items	EFA		CFA		
	Cronbach’s Alpha	Eigenvalue (variance explained)	Composite reliability	AVE	MSV
Factor 1: Convenience	0.89	12.88 (43.25)	0.89	0.59	0.52
CVN1	0.76		–		
CVN2	0.90		0.69		
CVN3	0.78		0.79		
CVN4	0.65		0.75		
CVN5	0.73		0.68		
CVN6	0.68		0.69		
Factor 2: Privacy security	0.90	1.98 (5.80)	0.90	0.70	0.32
PVT1	0.76		0.79		
PVT2	0.85		0.83		
PVT3	0.85		0.84		
PVT4	0.85		0.84		
Factor 3: Reliability	0.86	1.63 (4.42)	0.87	0.69	0.43
REL1	0.82		0.85		
REL2	0.79		0.75		
REL3	0.82		0.86		
REL4^a^	-		-		
Factor 4: Perceived value	0.86	1.53 (4.00)	0.86	0.60	0.52
VAL1	0.77		0.85		
VAL2	0.70		0.80		
VAL3	0.81		0.77		
VAL4	0.72		0.69		
Factor 5: Transaction cost	0.85	1.22 (3.11)	0.85	0.58	0.43
TCT1	−0.64		0.71		
TCT2	−0.57		0.76		
TCT3	−0.86		0.80		
TCT4	−0.84		0.69		
Factor 6: Customer satisfaction with Smart Locker	0.90	1.08 (2.61)	0.91	0.71	0.51
SSL1	0.75		0.90		
SSL2	0.64		0.79		
SSL3	0.98		0.86		
SSL4	0.84		0.83		
Factor 7: Customer satisfaction with E-commerce	0.90	1.06 (2.42)	0.90	0.70	0.51
SEC1	0.62		0.74		
SEC2	0.84		0.87		
SEC3	0.91		0.89		
SEC4	0.77		0.85		

CFA Model fit indices: Chi-square/df = 1.884; GFI = 0.907; CFI = 0.963; RMSEA = 0.045.

^a^Indicates that the observed variable has been removed from the model.

The overfit, reliability, and validity were evaluated using CFA. The standardised factor loadings, the composite reliability (CR), the average variance extracted (AVE), and the Maximum Shared Variance of the constructs are presented in Table 5. The fit indices of the measurement model presented at the bottom of Table 5 met the cut-off criteria specified by Hu and Bentler (1999), suggesting that the saturated model matched the market data. The CRs of the constructs were between 0.85 and 0.91, which were above the acceptable threshold of 0.70 (Hair Jr et al., 2010). This demonstrated that the internal consistency of the measurement items was reliable for representing their loaded constructs. The AVE of each construct was above the cut-off point of 0.50, and greater than the maximum shared variance (MSV), which ensured the convergent validity and discriminant validity of the measurement.

### Structural model analysis

**Fig. 2:**
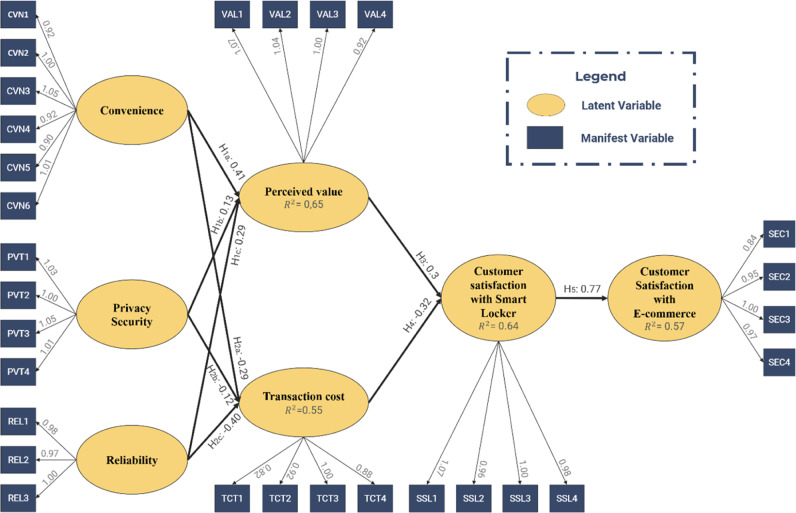
Structural equation model. Note: SEM model fit indices: Chi-square/df = 1.933; GFI = 0.905; CFI = 0.960; TLI = 0.956; RMSEA = 0.046.

Figure 2 shows the full structural model estimation of the research model using AMOS 20.0, with the model fit indices listed at the bottom demonstrating the appropriateness of this model. In addition, the *R* squared of endogenous variables (i.e., perceived value, transaction cost, customer satisfaction with Smart Locker, and customer satisfaction with e-commerce) were all greater than 0.5, which suggested that their exogenous variables explicated a larger part of the variance in the endogenous variables than the error terms.

As shown in Table 6, all hypotheses were accepted due to their *p*-value < 0.05. The positive relationships included (1) convenience and perceived value (*β* = 0.441); (2) privacy and security and perceived value (*β* = 0.337) (3) reliability and perceived value (*β* = 0.166); (4) perceived value and customer satisfaction with Smart Locker (*β* = 0.565); and (5) customer satisfaction with Smart Locker and customer satisfaction with e-commerce (*β* = 0.753). The negative effects included (6) convenience and transaction cost (*β* = −0.337); (7) privacy and security and transaction cost (*β* = −0.149); (8) reliability and transaction cost (*β* = −0.431); and (9) transaction and customer satisfaction with Smart Locker (*β* = −0.326).

**Table 6:** Standardised regression weights.

Hypothesis				*β*	S.E.	C.R.	*p*-value
H1a	CVN	→	VAL	0.441	0.055	7.426	***
H1b	PVT	→	VAL	0.166	0.037	3.468	***
H1c	REL	→	VAL	0.337	0.045	6.35	***
H2a	CVN	→	TCT	−0.293	0.062	−4.7	***
H2b	PVT	→	TCT	−0.149	0.044	−2.829	0.005
H2c	REL	→	TCT	−0.431	0.054	−7.291	***
H3	VAL	→	SSL	0.565	0.061	9.843	***
H4	TCT	→	SSL	−0.326	0.052	−6.207	***
H5	SSL	→	SEC	0.753	0.05	15.599	***

***Indicates that *p* < 0.001.

## Discussion

Using Smart Locker provides tangible benefits to users in the form of increased perceived value or reduced transaction costs. Perceived value and transaction costs can be improved by effective resources matching and through increasing the convenience, reliability, and security of Smart Lockers, thereby promoting customers’ intention to use this service (Yuen et al., 2019). However, this study went beyond investigating customers’ intentions (Yuen et al., 2019) and satisfaction (Tang et al., 2021) to explore the influence of customer satisfaction with Smart Locker on that with e-commerce platforms. This is expected to consolidate the contribution of last-mile delivery experiences to e-customers overall satisfaction.

Although the directions of the impact that convenience, reliability, and privacy and security had on perceived value and transaction costs completely aligned with the findings of Yuen et al. (2019), the levels of impact did not. The factor having the greatest influence on perceived value was reliability, as concluded by Yuen et al. (2019); however, it was convenience in this study. This could be attributed to the differences in the research context. Yuen et al. (2019) collected data from 10 cities in China with the highest number of online shopping transactions, where the Smart Locker service is well-developed and has wide coverage. The high level of competition among Smart Locker providers increases the standard of service; therefore, convenience is generally satisfactory, and customer interests are turned to reliability. In contrast, Smart Locker service in Vietnam has not been popular due to the limited quantity of available lockers. Furthermore, there are less opportunities for customers to choose this service due to the small number of e-commerce businesses adopting it. Consequently, convenience is the factor that has the strongest impact on customers’ perceived value.

Moreover, this study emphasises the convenience of Smart Locker locations rather than simply focusing on the technological and service aspects of Smart Locker, as done by Tang et al. (2021). This can also be attributed to the difference in research context between China and Vietnam. In Vietnam, the number of Smart Lockers is limited; therefore, we paid attention to the location of hubs when designing the convenience scale to provide better insights for providers.

## Conclusion

### Research implications

The importance of satisfaction has led to a large amount of research on this topic (Mittal and Frennea, 2010). In particular, the rapid development of e-commerce in recent years has encouraged studies on customer satisfaction. For instance, Vasić et al. (2019) identified determinants of customer satisfaction, including safety and quality of e-retailer websites, completeness of information on the websites, and cost and time spent on online purchases. Bakator et al. (2019) revealed the correlation between customer satisfaction and advertising. The present study contributed to current literature by exploring the impact of a specific last-mile delivery method on online customer satisfaction.

In addition, this study is one of the first to explore aspects of Smart Lockers that affect user satisfaction. Previous studies often focused only on technical factors, impact of using Smart Lockers on traffic, environment, economy, and customers’ acceptance and intention to use the service. Only recently, Tang et al. (2021) evaluated customer satisfaction with the experience, examining the influence of service quality aspects, including service price, diversity, reliability, convenience, and fault handling capability, on user satisfaction. However, Tang et al. (2021) examined users in China, which is a flourished e-commerce market and consumers are familiar with Smart Lockers. Conversely, the present study investigated the opposite context, that is, a developing e-commerce market with a low presence of Smart Lockers. Thus, research aspects were adjusted accordingly, contributing to a theoretical foundation for examining customer satisfaction with Smart Locker services.

### Managerial implications

This study explored a different approach to customer experience management of e-commerce businesses. The results indicated that customer satisfaction with the last-mile delivery service (i.e., Smart Locker) increased the level of satisfaction with their online shopping experience. This finding highlighted the important role of last-mile delivery, particularly Smart Lockers, in customer satisfaction. Thus, we suggest that e-commerce businesses consider integrating Smart Lockers into their delivery methods system. The convenience, reliability, and privacy and security of Smart Lockers enhance customers’ perceived value, thereby increasing the overall satisfaction with the online shopping experience. Furthermore, the application of Smart Locker as an automated solution that minimises human involvement can help e-commerce administrators comprehensively and objectively control, monitor, and evaluate customer experience at the last stage in the consumers’ online shopping journey.

Moreover, this study provides guidance for Smart Locker service providers to improve service quality and enhance user satisfaction. The results revealed three determinants that had a positive impact on customer satisfaction through the mediators of perceived value and transaction costs: convenience, reliability, and security. Therefore, service providers could focus on critical factors and develop effective measures to improve the service. Furthermore, businesses should focus on improving and optimising the value of convenience and reliability, as these factors are highly appreciated by customers and strongly influence satisfaction. With regard to convenience, providers could strategically place Smart Locker hubs to save users’ time and effort in accessing the locker (Collier et al., 2014; Lin and Hsieh, 2011; Roy et al., 2018). As for reliability, businesses should ensure that data processing systems operate accurately to eliminate the risk of miscommunication and problems with goods delivery. In addition, although security does not have as strong of an impact on user satisfaction, policies and measures to protect user information require attention to create a positive customer experience.

### Limitations and future research

This study had some limitations. First, the sample size was constrained due to limited time and financial resources. The authors purposefully selected people who used Smart Lockers on a voluntary basis to participate in the survey to discover the impact of using this service on online shopping behaviour. Although the sample size was adequate to meet the model requirements, further studies should increase sample size in more diverse contexts to generate more in-depth knowledge on the impact of Smart Locker use on online customer satisfaction.

Second, the demographic control variable was not considered comprehensively in our analysis. During the study period, Hanoi experienced a COVID-19 pandemic outbreak. Consequently, face-to-face interviews were only possible in green zones with low infection levels. Further analysis should include this variable in the structural model to explore its effect on Smart Locker use and online shopping behaviour.

Lastly, the COVID-19 pandemic is dramatically changing consumers’ shopping habits globally. The effects of these changes were not considered in this study. Future research is required on Smart Lockers and other emerging delivery models and their impact on consumer behaviour in the new context.

## Data Availability

Underlying data and syntax are available upon request from the corresponding author.
